# Concordance between Laboratory and Field Methods for the Assessment of Body Fat in Olympic Combat Athletes: Analysis of the Influence of Adiposity

**DOI:** 10.3390/ijerph19084493

**Published:** 2022-04-08

**Authors:** María Fernandez-del-Valle, Hugo Olmedillas, Nieves Palacios Gil de Antuñano, Ana María Ribas, Pablo Martínez-Camblor, Ángela García-Gonzalez, Natalia Úbeda, Eduardo Iglesias-Gutiérrez

**Affiliations:** 1 Department of Functional Biology, University of Oviedo, 33006 Oviedo, Spain; olmedillashugo@uniovi.es (H.O.); iglesiaseduardo@uniovi.es (E.I.-G.); 2 Health Research Institute of the Principality of Asturias (ISPA), 33011 Oviedo, Spain; 3 Sports Medicine Center, Higher Council for Sports, 28040 Madrid, Spain; nieves.palacios@aepsad.gob.es (N.P.G.d.A.); anaribasdieteticaynutricion@gmail.com (A.M.R.); 4 Department of Biomedical Data Science, Geisel School of Medicine at Dartmouth, Lebanon, NH 03756, USA; pablo.martinez.camblor@dartmouth.edu; 5 Department of Pharmaceutical and Food Sciences, CEU San Pablo University, 28668 Boadilla del Monte, Spain; angargon@ceu.es (Á.G.-G.); nubeda@ceu.es (N.Ú.)

**Keywords:** adiposity, body composition, elite athletes, methods agreement, Olympic combat sports

## Abstract

Combat sports athletes competing in the same discipline exhibit notable and substantial differences in body weight, body composition (BC) and adiposity. No studies have considered the influence of adiposity levels in the agreement between different BC assessment methods. The aim of this study was to analyze the influence of adiposity in the agreement between different methods used to estimate relative body fat (%BF) in Olympic combat sport athletes. A total of 38 male athletes were evaluated using air displacement plethysmography and dual-energy X-ray absorptiometry (DXA) as laboratory methods, and bioelectrical impedance analysis (BIA), near-infrared interactance (NIR) and anthropometry as field methods. All methods were compared to DXA. Agreement analyses were performed by means of individual intraclass correlation coefficients (ICCs) for each method compared to DXA, Bland–Altman plots and paired Student *t*-tests. The ICCs for the different methods compared to DXA were analyzed, considering tertiles of %BF, tertiles of body weight and type of sport. For the whole group, individual ICCs oscillated between 0.806 for BIA and 0.942 for anthropometry. BIA showed a statistically significant underestimation of %BF when compared to DXA. The agreement between every method and DXA was not affected by %BF, but it was highest in athletes at the highest %BF tertile (>13%). The ICC between NIR and DXA was poor in 72–82 kg athletes. Our results indicate that field methods are useful for routine %BF analysis, and that anthropometry is particularly appropriate, as it showed the highest accuracy irrespective of the athletes’ adiposity.

## 1. Introduction

Apart from high-level psychomotor skills and physiological capacities, other factors—particularly body composition (BC)—are critical for performance in combat sports [1]. Olympic combat athletes are classed for competition in body weight (BW) categories according to pre-competition weighing. Most often, these athletes use extreme BW cutting strategies prior to competing in order to fit into the most advantageous BW category (from 5 to 10% below their training BW) [2]. These strategies include inappropriate dietary practices involving severe and voluntary caloric restriction and active or passive dehydration [3]. These cause important changes in BC and hydration which can adversely affect performance in the short term [4]. Furthermore, these practices are considered an important health risk for the athlete [5] that could lead to athlete-triad-like symptoms, causing permanent health issues in the long term [6]. Therefore, it is highly important to maintain a BW that is close to the target competition BW throughout most of the season [7], with minimal fluctuations in fat, muscle and water content. For that reason, use of the most affordable, accurate and practical methods to monitor BC is of great importance and will help to control for BW variability, leading to optimal performance.

Several laboratory methods, such as underwater weighing, air displacement plethysmography (BOD POD), labelled water techniques and dual-energy X-ray absorptiometry (DXA) are considered reference methods to assess BC [8]. Among them, DXA has been suggested as one of the most appropriate methods for athletes, and it has been widely used as a reference method to validate other methods in this population [9]. BOD POD and DXA are expensive, non-portable methods, which makes them unsuitable for both field and, occasionally, repeated routine analysis. Field methods such as anthropometry, bioelectrical impedance analysis (BIA) or near-infrared interactance (NIR) seem more suitable for this purpose, although research has shown that they are generally less precise [9].

Anthropometry has been the main method used to assess BC in athletes due to its low cost and easy applicability. Its accuracy is high when the equipment is properly calibrated and the assessment is performed by a trained technician [9]. Recent studies employing BIA or DXA for the assessment of BC have shown that specific physique traits, such as lean mass, lean mass distribution and body mass index (BMI), in combat sport athletes differ by BW category [10,11].

The agreement between different methods used to assess relative body fat (%BF) has been widely analyzed in both the general population and in different sports [4]. Interestingly, it seems that the concordance between methods varies according to the adiposity content of the individuals assessed [12,13,14]. This is particularly important for combat sport athletes because differences in BW and BC can be substantial among those competing in the same discipline but in different BW categories [11]. Compared to DXA, BOD POD has been shown to overestimate %BF in thinner participants and underestimate %BF in heavier individuals [14]. Compared to DXA, BIA may overestimate fat mass and lean mass in men with different BC [12], underestimate %BF [15], offer an accurate estimate of %BF [16] or show different limits of agreement [17]. However, these studies were conducted with the general population, including normal weight, overweight and obese male individuals [12,14,15,16,17]. In a study conducted with healthy active males, NIR showed a good concordance with DXA, but tended to overestimate %BF in leaner subjects and underestimate %BF in those with a higher %BF [13].

To the best of our knowledge, no studies have considered the influence of adiposity levels in the agreement between different methods, nor included a comprehensive selection of field and laboratory methods for %BF assessment in combat sport athletes.

Research that compares the agreement between field and laboratory methods for more efficient maintenance and improvement of combat sport athletes’ performance is highly relevant for coaches and sport medicine professionals. Therefore, the main objective of this study was to examine the agreement between field and laboratory methods compared to DXA in a group of Olympic combat sport athletes, considering the influence of adiposity as a potential confounding variable.

## 2. Materials and Methods

This study used a cross-sectional observational design. To be eligible, athletes had to be free of injuries or not in recovery, participating in regular training and not engaging in weight loss practices. The study was conducted according to the guidelines of the Declaration of Helsinki and was approved by the Research Ethics Committee of the Principality of Asturias (protocol code ID: 46/16; 4 December 2016). Informed consent was obtained from all subjects involved in the study.

Sixty-one male combat sport athletes from national teams were contacted to participate in this study. A total of 38 athletes (Taekwondo, *n* = 12; Judo, *n* = 10; Wrestling, *n* = 10; Boxing, *n* = 6), including one Olympic medalist and one World champion, signed a written informed consent form.

Athletes were assessed one week prior to an international competition. All data for each athlete were collected the same morning, in a fasted state of at least 8 h, starting at 8 am (<2 h). Relative body fat was assessed using laboratory and field methods. Laboratory methods included a DXA scan (Norland XR-46, Norland Co., Lincoln, NE, USA) and BOD POD (Cosmed, Rome, Italy). DXA was considered the reference method [9]. As field methods, BIA (Tanita BC-418, Tanita Corporation of America, Inc., Arlington Heights, IL, USA), NIR (Futrex-6100, Futrex, Gaithersburg, MD, USA) and anthropometry (Seca 769 scale with stadiometer, Seca Corporation, Hamburg, Germany; Holtain caliper, anthropometric tape and anthropometer, Holtain, Crymych, UK) were used. Standard techniques were used for all methods, as described elsewhere [18]. Regarding anthropometry, data were collected following the International Society for the Advancement of Kinanthropometry (ISAK) standards [19] by the same ISAK Level-3 anthropometrist. Anthropometry measurements included ISAK-Restricted Profile, that is, body mass, height, 8 skinfolds, 5 circumferences and 2 bone breadths. Body mass index was calculated as BW in kilograms divided by the square of the height in meters (BMI = kg/m^2^).

The Siri equation [20] was used to estimate %BF from body density determined by DXA [21] and BOD POD. The equation of Kyle et al. [22] was used to estimate %BF based on the information obtained from the multifrequency bioimpedance device. In order to estimate %BF, the optical densities (ODs) at specific NIR wavelengths (810 and 944 nm) were recorded. Finally, %BF for anthropometry was estimated using Evan’s equation [13].

Continuous variables were summarized by mean and standard deviation (SD). Equality of means by groups were compared using the robust Welch test. Standard statistical analyses for assessing agreement [23] were used. These analyses included intraclass correlation coefficients (ICCs), where we reported individual and mean coefficients as well as 95% confidence intervals, Bland–Altman plots, paired means comparison and paired Student’s *t*-tests were used, and 95% confidence intervals for the mean differences were reported. Additionally, a subgroup analysis was performed and the results were summarized in a forest plot. Tertiles of %BF analyzed with DXA, tertiles of BW and type of sport were described in order to analyze the possible influence of these variables on the agreement between all methods compared to DXA. Although we realize that there are established groups of BW in these sports, because of the sparse sample sizes available, the tertiles categorization was chosen in order to optimize the statistical power within each group. All statistical analyses were performed using the software R (R Foundation for Statistical Computing, Vienna, Austria).

## 3. Results

Table 1 shows age, BW, height and BMI, as well as %BF derived from DXA, BOD POD, BIA, NIR and anthropometry assessment for the whole group, separated by sports. Significant differences in height and NIR-derived %BF were observed when comparing sports. Taekwondo athletes were significantly taller than Wrestling and Boxing athletes (*p* = 0.010), while Judo athletes showed a significantly higher adiposity in NIR-derived %BF compared Taekwondo and Boxing athletes (*p* = 0.011).

Individual ICCs for each method compared to DXA-derived %BF oscillated between 0.806 for BIA and 0.942 for anthropometry, as shown in Table 2. The ICC values were larger for means, ranging between 0.892 (BIA) and 0.970 (BOD POD and anthropometry). BIA showed a statistically significant underestimation of %BF when compared to DXA. No other relevant differences were found between the observed means.

**Figure 1:**
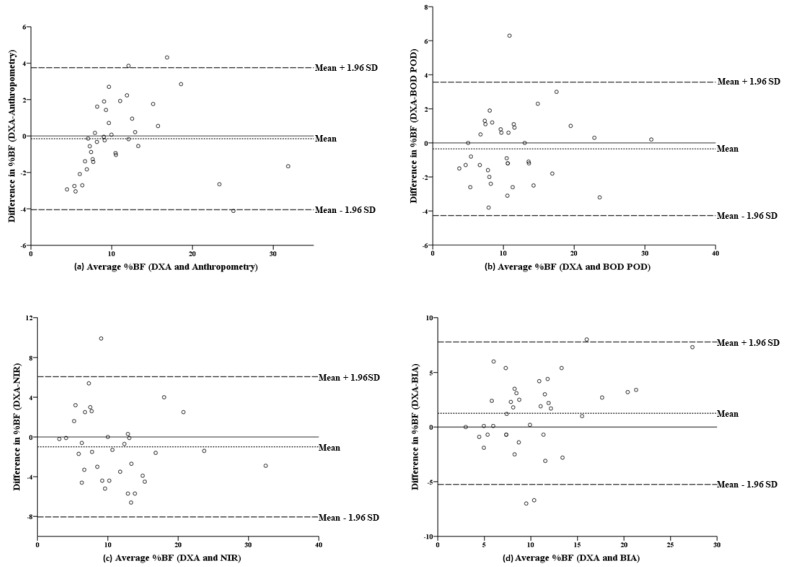
Bland–Altman plots representing the difference between DXA-derived %BF and %BF determined using (**a**) anthropometry, (**b**) BOD POD, (**c**) NIR and (**d**) BIA. The central dotted line represents the mean inter-methods difference. The upper and lower broken lines represent the 95% limits of agreement (inter-methods difference ± 1.96 SD of the differences). Abbreviations: %BF = percentage of body fat; DXA = dual-energy X-ray absorptiometry; BOD POD = air displacement plethysmography; NIR = near-infrared interactance; BIA = bioelectrical impedance analysis; SD = standard deviation.

The Bland–Altman plots for each method in reference to DXA (Figure 1a–d) show an average line very close to zero and most of the points within the confidence levels, which indicates a good agreement. Anthropometry was reported as the most consistent method when assessing %BF (Figure 1a). Agreement was reduced in low %BF combat athletes in all methods.

**Figure 2:**
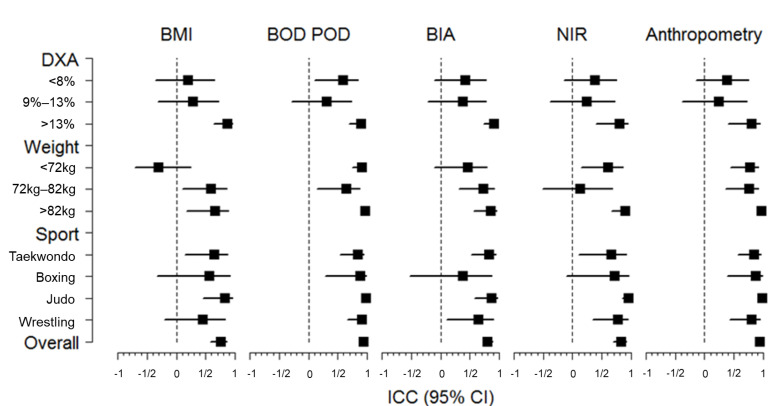
Forest plot: ICC (crude) and 95% confidence interval for the relationship between BMI and %BF (measured by BOD POD, BIA, NIR, and anthropometry) by subgroups (sport) or tertiles (%BF measured by DXA and weight). Abbreviations: ICC = intra-class correlation; CI = confidence interval; BMI = body mass index; %BF = percentage of body fat; BOD POD = air displacement plethysmography; NIR = near-infrared interactance; BIA = bioelectrical impedance analysis; DXA = dual-energy X-ray absorptiometry.

Figure 2 depicts the ICCs (with a 95% confidence band) of the different methods compared to DXA, considering the overall group as well as several subgroups: tertiles of %BF analyzed by DXA, tertiles of BW and type of sport. Although robust results were reported for most of the subgroups considered, we observed that the ICCs between all methods and DXA were highest in athletes with higher %BF (>13%). Interestingly, the ICC between NIR and DXA in athletes with BW 72–82 kg was poor. No relevant differences were observed among sports.

## 4. Discussion

This is the first study to analyze the concordance between DXA, as a reference method, and other methods for the estimation of %BF in a sample of elite male Olympic combat sport athletes prior to an international competition. Our results showed a good agreement between DXA and field methods, although anthropometry—performed by a certified technician—showed the highest concordance. Adiposity did not influence the observed concordance between methods since no trend was observed in the Bland-Altman plots. Furthermore, subgroup analyses of %BF tertiles confirmed that these results are robust. Particularly, ICCs were higher in the DXA group with %BF > 13% for all methods, indicating that the observed differences between DXA and the different methods are constant in absolute value. Findings from this study have strong implications for combat sport athletes, as using the most accurate and practical methods for routine assessment is essential in order to optimize training periodization. Additionally, our results suggest that there may be multiple methods, rather than only one, that could be useful for regular evaluations. However, not all methods behaved the same.

A good agreement was observed in the present study when analyzing BOD POD and DXA-derived %BF (individual ICC = 0.941, 95% CI: [0.889; 0.969]). Contrary to our results, Collins et al. [24] found that %BF obtained using BOD POD was smaller than DXA-derived %BF values in a sample of 20 collegiate American football players. However, this study showed a substantial difference in %BF variability (10.7 to 23.5%) that was not considered in the agreement analysis [24]. Supporting our findings, Santos et al. [10] reported that DXA overestimated %BF at lower ends in elite male judokas when compared to a four-compartment model and showed large limits of agreement ranging from −3.7 to 5.3. Utter et al. and Bentzur et al. showed that BOD POD provided similar %BF values compared with hydrostatic weighing (in young male wrestlers) [25] and DXA (in female track athletes) [26], respectively. The low standard error of the estimate and the high adjusted R^2^ obtained from the regression analysis highlight the value and suitability of this method as an alternative in this population [25]. Using BOD POD might not be the most practical alternative as a routine method for the assessment of adiposity levels, compared to field methods. Nevertheless, further research in larger samples and multiple sports would be necessary in order to evaluate its utility as a reference method for %BF assessment in different disciplines.

Anthropometry is one of the most widespread methods for the assessment of BC in high-performance athletes [27]. However, research investigating the concordance of anthropometry and DXA-derived %BF in combat sport athletes is lacking. In our study, anthropometry-derived %BF showed the best agreement with DXA (individual ICC = 0.942, 95% CI: [0.891; 0.969]) among all studied methods. Our study showed a higher mean value of anthropometry-derived %BF (14.3%) for judokas compared to the results (7.8%) reported by Drid et al. [28]. This large difference could be explained by differences in the samples recruited, that is, all BW categories versus only half-heavyweight medalists, respectively. Furthermore, the equation used for the calculation of %BF was not reported by the authors [28]. In our study, we used Evans’ equation, which was designed specifically for athletes and was based on a four-component model—the best predictor of BC [29]. Silva et al. [30] observed that Evan’s equation overestimated adiposity when compared to a four-compartment model of %BF that included DXA, BIA and BOD POD (9% vs. 7%) in judokas. They reported that Evan’s equation (9%) overestimated adiposity compared to a four-compartment model of %BF (7%) that included DXA, BIA and BOD POD methods. Based on our results, we could conclude that anthropometry might be the most suitable method for the assessment of adiposity, considering it is portable and economical. However, unlike other field methods, anthropometry largely relies on the technical ability of the technician, which is a determinant of the accuracy and precision of the measurements [31].

Despite the high agreement with DXA-derived %BF observed for NIR, it was lower than the agreement found for anthropometry, or tended to underestimate (Taekwondo) or over-estimate (Judo) %BF. The concordance with DXA was affected by adiposity and BW, as previously reported by Evans et al. [29]. This study found that NIR underestimated %BF in a group of individuals who exercise regularly, while it overestimated %BF in another group with lower BW who perform resistance training. Therefore, NIR does not seem to be the most appropriate method for disciplines in which athletes show important differences in BW and BC.

Despite showing a good agreement, the lowest concordance with DXA was reported for BIA-derived %BF (individual ICC = 0.806, 95% CI: [0.657; 0.894]). The %BF estimated by BIA was 6–24%, which was lower than DXA in all groups, except boxers, for which it was 10% higher. Interestingly, boxers were older, shorter, heavier and leaner compared to athletes in the other subgroups. These differences were only statistically significant for height. Additionally, only six boxers from different BW categories were included in the present study. Thus, no robust statistical analysis could be performed. A weak agreement between BIA- and DXA-derived %BF has been observed by other authors studying collegiate male baseball players [32], water polo, judo and karate male athletes [33], and elite soccer and ice hockey players [34]. These authors indicated a lack of control of confounding variables, particularly hydration status and recent strenuous exercise, that may have explained their results. In the present study, all participants followed a standardized measurement protocol to account for potential confounding variables, but we still found a poor agreement with DXA. Our results for BIA are also in agreement with those of Drid et al. [28], who evaluated elite and sub-elite European half-heavyweight male judokas. These athletes had a similar %BF compared to those reported in this study [28]. Other research utilizing BIA as a reference method found large limits of agreement with other techniques, which resulted in significant overestimations and underestimations [4]. Thus, BIA has been proposed as an instrument to evaluate hydration level in combat sport athletes [35], however, other researchers question its validity for this purpose [36]. Furthermore, Hetzlet et al. [37] analyzed the concordance between BIA and anthropometry in wrestlers and concluded that they are not to be used interchangeably. These results are in agreement with our findings, since ICC was 0.970 for anthropometry-derived %BF but was lower (0.892) for BIA than for DXA. This highlights the relevance of anthropometry as an appealing field method for %BF measurement in combat sport athletes, especially compared to other field methods like BIA.

Although DXA has become the gold standard for bone mass, it has been suggested as a reference for the soft tissue assessment of fat mass and percentage body fat [38]. However, the differences between devices, manufacturers and software versions can lead to divergent results [39]. For our study we utilized the same equipment, protocols, procedures and the same technician for all participants. Another limitation relates to the use of assessment methods with the assumptions associated with the two-compartment model. One of the main problems is the assumption that total body water does not vary. In our study, we monitored hydration levels, and the athletes engaging in weight loss practices were excluded from the study. The sample size of this study presents another limitation. A greater sample size would allow for multiple analyses, including other adiposity indices such as fat mass, fat mass index or fat distribution. However, the potential number of elite combat athletes that can be approached to participate is small, and their availability is limited. Future studies may overcome this limitation by conducting multicentric international studies, or by including non-elite athletes. Nevertheless, this last approach will impact the BC profiles of athletes, which may affect accuracy. Another possible limitation is that measurements were taken only once (except anthropometry, which was taken in duplicate), although all assessments were performed in the same session. It would have been desirable to have repeated measurements. According to our experimental design, measurements were all taken at the same timepoint in order to avoid the influence of common intra-seasonal variability in BW and %BF. These strict methodological criteria made it difficult to perform more than one measurement. Additionally, all the methods used to estimate %BF in this study show a high reproducibility and accuracy, as previously tested [40].

## 5. Conclusions

According to these results, anthropometry, NIR and BIA are suitable field methods to determine %BF in elite male combat sport athletes, when properly applied and with an adequate selection of equations. Furthermore, adiposity did not influence the observed concordance. Anthropometry showed the highest level of concordance, without differences among athletes with various adiposity levels, making it suitable for any BW category. Therefore, anthropometry—when performed by a trained technician—is particularly appropriate, although it relies largely on the technical skills of the anthropometrist. Among laboratory methods, BOD POD arises as an alternate routine method for %BF assessment in this population. It would be necessary, however, to extend this study to a larger sample in order to identify accuracy and concordance thresholds of adiposity for combat sports in general and within sporting disciplines.

## Figures and Tables

**Table 1:** Descriptive analysis and *p*-values for combat sports athletes’ %BF—total and by sport—measured using laboratory and field methods.

Variables	Total (*n* = 38)	Taekwondo (*n* = 12)	Judo (*n* = 10)	Wrestling (*n* = 10)	Boxing (*n* = 6)	*p*
Age (yr)	20.3 ± 3.6	19.8 ± 4.1	19.9 ± 2.8	20.0 ± 3.2	22.3 ± 4.4	0.678
Weight (kg)	78 ± 17	78 ± 9	77 ± 9	77 ± 9	88 ± 25	0.156
Height (m)	1.79 ± 0.07	1.84 ± 0.04 *	1.81 ± 0.08	1.76 ± 0.06	1.73 ± 0.08	0.010
BMI (kg·m^−2^)	24.2 ± 3.9	23.1 ± 2.5	26.6 ± 5.6	24.7 ± 2.1	21.4 ± 3.4	0.093
DXA (%)	11.0 ± 5.9	11.4 ± 5.0	12.8 ± 9.2	10.7 ± 3.2	7.8 ± 3.4	0.273
BOD POD (%)	11.5 ± 5.8	10.9 ± 4.6	13.7 ± 9.2	10.8 ± 2.5	9.5 ± 4.2	0.724
BIA (%)	9.8 ± 4.7	8.7 ± 4.2	12.0 ± 6.5	9.7 ± 3.3	8.3 ± 3.4	0.489
NIR (%)	11.7 ± 6.5	8.6 ± 4.1	15.2 ± 8.4 ^#^	14.1 ± 3.4	6.5 ± 5.4	0.011
Anthropometry (%)	11.2 ± 5.8	10.8 ± 3.3	14.3 ± 9.8	10.1 ± 2.6	8.5 ± 2.4	0.227

* p < 0.05, comparing Taekwondo with Wrestling and Boxing; ^#^ *p* < 0.05, comparing Judo with Taekwondo and Boxing. Abbreviations: %BF = percentage of body fat; BMI = body mass index; DXA = dual energy X-ray absorptiometry; BOD POD = air displacement plethysmography; BIA = bioelectrical impedance analysis; NIR = near-infrared interactance.

**Table 2:** Percentage of body fat mean, and individual and mean difference intraclass correlation coefficients with a 95% confidence interval for the different methods compared to DXA.

Method	Mean Dif. (95% CI)	ICC (95% CI)		
		Individual	Mean	*p*
Anthropometry	−0.15 (−0.80; 0.51)	0.942 (0.891; 0.969)	0.970 (0.943; 0.984)	0.624
BOD POD	−0.35 (−1.02; 0.32)	0.941 (0.889; 0.969)	0.970 (0.941; 0.984)	0.293
NIR	−0.99 (−2.22; 0.25)	0.833 (0.694; 0.912)	0.909 (0.820; 0.954)	0.114
BIA	1.26 (0.17; 2.35)	0.806 (0.657; 0.894)	0.892 (0.793; 0.944)	0.025 *
BMI	−13.2 (−14.3; −12.0)	0.755 (0.576; 0.864)	0.860 (0.731; 0.927)	<0.001 **

* *p* < 0.05, comparing BIA with DXA values; ** *p* < 0.01, comparing BMI with DXA values. Abbreviations: DXA = dual energy X-ray absorptiometry; BOD POD = air displacement plethysmography; NIR = near-infrared interactance; BIA = bioelectrical impedance analysis; BMI = body mass index; ICC = intraclass correlation coefficient; CI = confidence interval.

## Data Availability

Due to ethical concerns, supporting data cannot be made openly available. The data that support the findings of this study are available upon request from the authors.
